# TRANSGENDERED OLDER ADULTS IN LONG-TERM CARE: PREPARING FOR THE RISING TIDE

**DOI:** 10.1093/geroni/igz038.2538

**Published:** 2019-11-08

**Authors:** Jean Henry, Susan K Patton

**Affiliations:** University of Arkansas, Fayetteville, Arkansas, United States

## Abstract

Gender non-conforming older adults are more likely to be without traditional support systems in place; many may need to turn to nursing homes for long-term care (LTC). Little information is available about the experiences of LGBT older adults in these settings. Research questions: What is known about the experiences of transgender (TG) older adults in LTC? What evidence-based recommendations exist to guide LTC administrators in providing quality care to TG residents? What are key future research areas? Method: Systematic review of extant literature, using databases: PubMed, Medline, Psych Info, CINAHL, Academic Search Complete, and ProQuestCentral. Key Results:RQ1) Published research in transgender healthcare consists primarily of case reports, and retrospective and cross-sectional studies; minimal literature specifically on TG individuals; combining LGBT carries risk of minimizing or equating the TG experience to that of gays and lesbians. RQ2) Recommendations: take proactive approach; focus on awareness of relevant laws and regulations; establish non-discriminatory and inclusive environments, policies, and procedures; regular staff training, monitoring, and evaluation. RQ3) Need exists for quantitative and qualitative research into all aspects of the TG experience in LTC. Key areas include: lived experience of the TG in LTC; beliefs, attitudes, values and practices of LTC staff; administrative challenges and responses. Conclusions: Transgender and gender non-conforming individuals frequently experience discrimination in healthcare. Lack of cultural and clinical competence, and discrimination and bias by providers, create barriers to quality healthcare. Healthcare professionals and systems must be proactive in preparing for the inevitable growth of this population in the LTC setting.

